# VectorBase: an updated bioinformatics resource for invertebrate vectors and other organisms related with human diseases

**DOI:** 10.1093/nar/gku1117

**Published:** 2014-12-15

**Authors:** Gloria I. Giraldo-Calderón, Scott J. Emrich, Robert M. MacCallum, Gareth Maslen, Emmanuel Dialynas, Pantelis Topalis, Nicholas Ho, Sandra Gesing, the VectorBase Consortium, Gregory Madey, Frank H. Collins, Daniel Lawson

**Affiliations:** 1 Department of Biological Sciences, University of Notre Dame, Notre Dame, IN 46556, USA; 2 Department of Computer Science and Engineering, University of Notre Dame, Notre Dame, IN 46556, USA; 3 ECK Institute for Global Health, University of Notre Dame, Notre Dame, IN 46556, USA; 4 Department of Life Sciences, Imperial College London, South Kensington Campus, London SW7 2AZ, UK; 5 European Molecular Biology Laboratory, European Bioinformatics Institute (EMBL-EBI), Wellcome Trust Genome Campus, Hinxton, Cambridge CB10 1SD, UK; 6 Institute of Molecular Biology and Biotechnology (IMBB), FORTH, Vassilika Vouton,Nikolaou Plastira 100, 70013 Heraklion, Crete, Greece; 7 Center for Research Computing, University of Notre Dame, Notre Dame, IN 46556, USA

## Abstract

VectorBase is a National Institute of Allergy and Infectious Diseases supported Bioinformatics Resource Center (BRC) for invertebrate vectors of human pathogens. Now in its 11th year, VectorBase currently hosts the genomes of 35 organisms including a number of non-vectors for comparative analysis. Hosted data range from genome assemblies with annotated gene features, transcript and protein expression data to population genetics including variation and insecticide-resistance phenotypes. Here we describe improvements to our resource and the set of tools available for interrogating and accessing BRC data including the integration of Web Apollo to facilitate community annotation and providing Galaxy to support user-based workflows. VectorBase also actively supports our community through hands-on workshops and online tutorials. All information and data are freely available from our website at https://www.vectorbase.org/.

## INTRODUCTION

VectorBase is a National Institute of Allergy and Infectious Diseases (NIAID) and National Institutes of Health (NIH) funded Bioinformatics Resource Center (BRC) (1). Our mission is to support the invertebrate vector research community by providing access to genome assemblies, genome annotations and high-throughput genomics data. For example, VectorBase is often involved in the first-pass annotation of *de novo* genome assemblies and subsequent capture of community annotations. More recently, genome variation, gene expression and proteomics were integrated with respect to reference genomes, as well as non-genic data such as field-associated samples from surveillance studies, insecticide-resistance phenotypes and pathogen transmission data. Controlled vocabularies and ontologies are used to organize and annotate experimental and sample-related metadata where appropriate.

VectorBase now hosts the genomes from 35 organisms: 22 mosquitoes (*Aedes aegypti*, *Culex quinquefasciatus* and 20 *Anopheles spp.* including major, minor and non-vectors), tick (*Ixodes scapularis*), body louse (*Pediculus humanus*), kissing bug (*Rhodnius prolixus*), 5 tsetse flies (*Glossina spp.*), house fly (*Musca domestica*), 2 sandflies (*Lutzomyia longipalpis* and *Phlebotomus papatasi*) and the intermediate snail host of S*chistosoma mansoni*, *Biomphalaria glabrata*. In the future, we anticipate hosting other important vector genome clusters such as the blackflies (*Simulium spp*). Current details about genomes hosted by the BRC can be found at https://www.vectorbase.org/genomes.

After the 2002 sequencing of the *Anopheles gambiae* genome, NIAID/NIH requested development of a BRC that would host data on that *Anopheles* genome and all newly sequenced genomes of invertebrate vectors that transmit human pathogens. The VectorBase BRC was proposed in response to that request and was first funded in June 2004 under a broader initiative entitled ‘BRCs for Biodefense and Emerging/Re-emerging Infectious Diseases’. That initiative had special focus on organisms in the NIAID Category A–C priority pathogens, i.e. those causing emerging and re-emerging diseases, and for VectorBase, the vectors transmitting such diseases. All VectorBase source code is made publically available and can be obtained either from online repositories or by contacting the BRC. With VectorBase now entering its second decade, usage by the scientific community has grown significantly (per Google Analytics) and its user community (per Google Scholar) has cited or acknowledged VectorBase in over 1230 publications.

## NEW FEATURES

### Website

As the project has expanded, the site's navigation focus has shifted to data types rather than a small set of reference genomes. Hence, the BRC now contains prominent links to genomes, transcriptomes, proteomes, mitochondrial sequences and population biology data. Other aspects of the site have been re-organized to improve navigation. For example, organism pages are now dynamically generated depending on the available data and tools for each organism. Many of these changes were achieved by adopting the popular Drupal content management system, which facilitates the rapid inclusion of new features while maintaining internal consistency of tables, summaries and pages. Drupal also provides users with personal accounts for enhancing their use of the BRC, and past analyses like Basic Local Alignment Search Tool (BLAST) jobs can now be securely retrieved, along with user-specific tool parameterization. The site continues to use cookies to store data relating to the genome browser such that users can configure evidence tracks that are on/off by default.

### Accessing data: search

**Figure 1.**
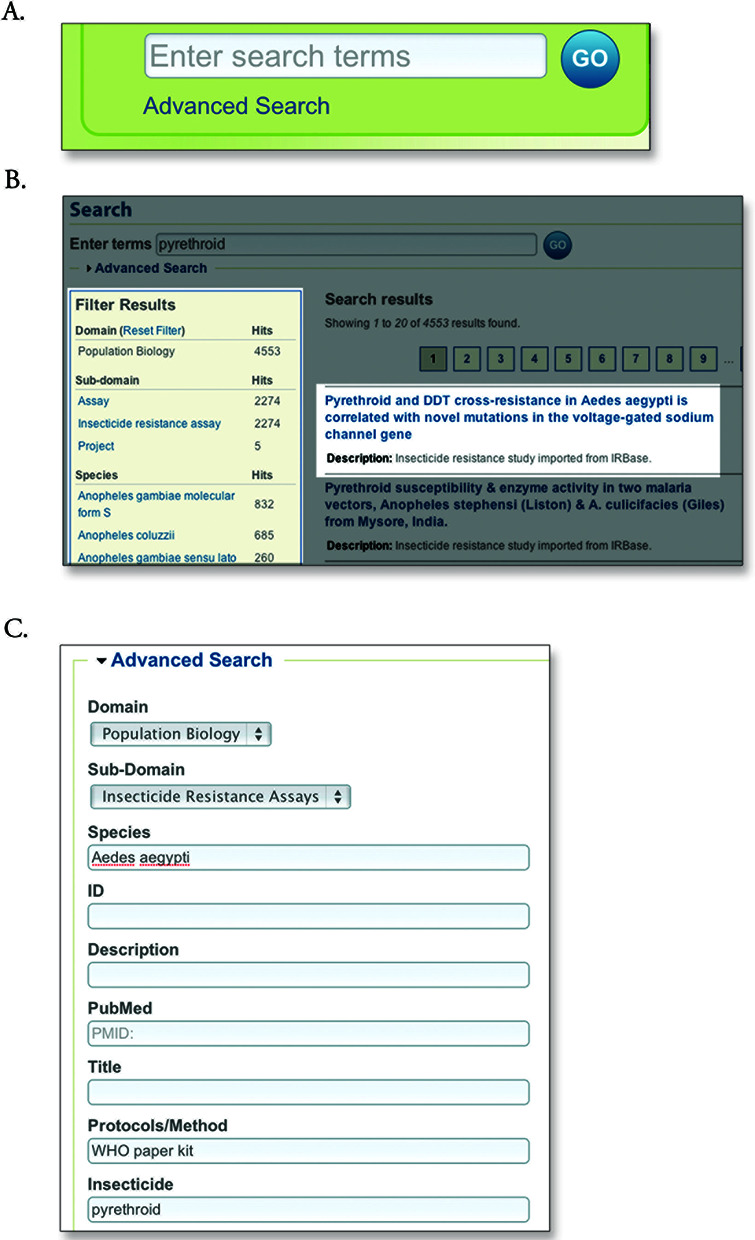
A search workflow to find VectorBase data. (**A**) The search box is located in the top right-hand side of all VectorBase pages. Keywords for the queries can be typed in the box or the user can select to go to ‘Advanced Search’. (**B**) The word ‘pyrethroid’ could retrieve a results page like this one (Because VectorBase data is updated every 2 months, you may obtain different results). In the left-hand side is the ‘Filter Results’ box, the results can be filtered down when clicking on the domains, subdomains or species links. The filters can also be reset. In the center of the page are the results, linking to the page where they are displayed. (**C**) Alternatively, advanced search can be used to start with a more specific query, making use of the different menus and boxes.

Accompanying the website's shift to data types, VectorBase reimplemented a data-centric search system powered by Apache Solr (Lucene Solr, http://lucene.apache.org/solr/#intro). All VectorBase release data are now organized into domains (e.g. Genome, Gene expression, PopBio) and subdomains (e.g. Genes, Samples, Assays), which allows efficient filtering of diverse results via a new faceted search feature. Simple keyword searches are available from every page on the website (Figure 1), while more complex and/or large-scale queries to search and retrieve data can be conducted using the BioMart system (2). The search system is under active development in two main areas. First, we are expanding the availability of ‘ontology enhanced’ search from the currently available PopBio domain to all domains. Ontology-enhanced search enables users to query for broad-scale concepts such as ‘insecticide’ and retrieves results for items annotated with fine-scale-related concepts such as ‘permethrin’ and ‘DDT’ (dichlorodiphenyltrichloroethane). This is achieved through the annotations of items in our databases with ontology terms developed within and outside VectorBase. Second, advanced search, which facilitates complex field-based queries, is being further developed to facilitate specific-use cases obtained from our Scientific User Group to improve overall effectiveness of search for our users.

### Gene manual annotations and metadata

VectorBase generates gene sets for each organism based on aggregating both *ab initio* and evidence-based gene predictions using the MAKER system (3). Although gene prediction is influenced greatly by available transcript evidence and tandemly arrayed gene clusters in the genome, our BRC is further affected by our diverse taxonomic breadth where some genomes have many informative comparators (e.g. mosquitoes) while others such as a tick or gastropod have relatively little data available. To address these issues, VectorBase has adopted the Web Apollo tool (4) where users can immediately view and modify annotations for inclusion into subsequence releases of the canonical gene set. For the submission of gene metadata, PubMed citations, repeats or RNA-seq experiments, we provide a separate submission interface based on forms.

### Galaxy

Galaxy is an open web-based science gateway that facilitates access to a wide range of computational tools (5). It is widely used for bioinformatics analysis with an extensive developer and outreach support network, and the web-based interface allows providing common tools in a transparent and reproducible framework. As tools can be connected in an intuitive way in so-called workflows, users can also easily manage their data, share analysis pipelines, and edit and re-run jobs/workflows without locally installing software. VectorBase has also made available the latest relevant canonical data in this Galaxy instance. Examples of workflow analyses include alignment of Next Generation Sequence (NGS) data sets, calculation of expression values, or predicting single nucleotide polymorphisms (SNPs). Registered users can store their raw data, intermediate files and final analysis results for download or direct sharing via the BRC, which greatly improves the ability to collaborate with colleagues and VectorBase developers.

### Expression map

The expression resource has grown over the years to contain a wide variety of quantitative expression data, particularly for the main mosquito vectors *A. gambiae* and *A. aegypti*. Gene expression report pages provide an overview of a gene's expression in many experiments, but the transcriptional regulatory context of that gene in relation to all others was lacking. The expression map (6) was developed to visualize the clustering of all genes of an organism based on their combined expression profiles from a large number of experiments (excluding a few with excess missing data or noise). The clustering is performed using a self-organizing map algorithm using a grid of 25 × 20 clusters. The web interface allows the user to explore and infer the function of genes in a particular cluster or neighborhood, to find genes with particular expression characteristics, and to give a functional overview of large gene families (7). It is integrated with BioMart for advanced data retrieval and further query refinement.

### Ontology browser

We have developed a new lightweight ontology browser with extended web browser compatibility (Chrome, Internet Explorer, Firefox, Opera and Safari). This is, to our knowledge, the only web browser-based ontology browser that is capable of efficiently handling very large ontologies (e.g. Gazetteer ∼530 000 terms) with fast response times. The root terms of the selected ontology are displayed in a tree fashion and the user can expand or collapse branches/nodes. The ontology browser has a powerful search capability for terms, including their synonyms, as well as partial and misspelled ones. Moreover, in the case of ontologies in which images are linked to terms (e.g. the anatomy ontologies in VectorBase), the browser is also able to display those. The latest functionality added to the ontology browser is the integration of VectorBase's search engine. Upon clicking on a term on the ‘tree-output’ of the ontology-browser, a search request is posted to the server and the returned results are displayed in a panel next to the ontology tree. These are the same results that would have been shown if one had directly searched VectorBase using the term's name or its ID as a query.

## NEW DATA

### Population biology/insecticide resistance

As detailed in our previous update (8), a population biology (PopBio) browser prototype was introduced in 2011 in anticipation of receiving increased amounts of genome-scale data from field, population and insecticide-resistance studies. The PopBio browser provides structured metadata for samples, assays, genotypes and phenotypes and the projects/publications to which they belong, supplemented by more detailed genetic variation data displayed in the genome browser where appropriate. Advanced visualization and meta-analysis tools are still under development but the PopBio browser already provides basic summary visualizations for nearly all projects, including plots of numeric data, and for categorical data, histograms and zoomable/scrollable maps with automatically adjusting pie-charts are shown.

A major overhaul of the PopBio infrastructure was undertaken in 2012–13 and a few of the more user-visible changes are described here. URL robustness and data maintainability were improved by the allocation of stable IDs for projects, samples and assays. The submission procedure was streamlined to a single ISA-Tab (9) spreadsheet submission. When using Google Spreadsheets, the user has direct access to an ontology term lookup tool called OntoMaton (10) and the ability to fill in data collaboratively with colleagues or seek assistance from VectorBase curators. The PopBio and genome browsers are now integrated via mutual links for samples with high-throughput genotype assays.

As of the August 2014 release, the PopBio resource contains 57,312 samples from 99 projects, of which 81 are insecticide-resistance studies imported from IRBase (8). A full list is available in the PopBio browser under the Tools menu, and only a few notable additions are described here. To supplement the large multi-country UCLA/UC Davis (University of California, Los Angeles / University of California, Davis) data set of *A. gambiae* species complex chromosomal inversion karyotypes that was present in the prototype, two similar data sets for Burkina Faso and Cameroon from other research groups have been added (11,12). This has expanded coverage of this rapidly evolving malaria vector both spatially and temporally. More recently, we developed a ‘meta project’ (PopBio project VBP0000012) that stores 8496 samples collected in Cameroon by three different research teams over a period of four years. Several thousand field-isolate species identifications used in the Malaria Atlas Project (13) covering four continents are also now available in the BRC. A large-scale insecticide-resistance phenotyping and genotyping study (14) has also been incorporated (VBP0000004) with genome variation browser integration. After October 2014, PopBio will become the NIH NIAID-supported insecticide-resistance repository for the vector community and will be open for new data submissions. Advanced search has a specialized ontology-enhanced subdomain for insecticide-resistance data.

### Genomic variation

We significantly expanded variation data sets for both new and previously described species since our last update. VectorBase currently contains genetic variation data for 11 disease vector species (nine *Anopheles*, one *Aedes* and *Ixodes*), with most species having in the range of 1–10 million SNP and indel variants. The functional effects of DNA variants on transcripts are calculated for all species each release, and the availability of the Ensembl Variation Effect Predictor (15) allows users to evaluate their own novel SNP and indel variant effects and display the results in their genomic context within the browser. Where possible, variants are linked back to their originating studies and phenotypes, and support for basic population genetic data such as allele frequencies and linkage disequilibrium calculations is provided.

Linkage of genomic, phenotypic (e.g. insecticide resistance) and population genetic data is also being developed through the PopBio browser system that provides search tools and geolocation displays of sample data, along with cross-linkage back to the underlying molecular variants. We also intend to integrate large-scale population sampling data published by the community to provide a consolidated resource for researchers to compare studies using the BRC.

### Ontologies

VectorBase continues to develop and maintain ontologies relating to control of disease vectors (16). Specifically, we host anatomy ontologies (TGMA -Taxonomist Guide of Mosquito Anatomy for mosquitoes and TADS – Tick Anatomy Daniel Sonenshine- for ticks (17)) and an ontology of insecticide resistance (MIRO - Mosquito Insecticide Resistance Ontology (18)). Our most recent additions are (i) a new ontology describing dengue fever (IDODEN – Infectious Disease Ontology DENgue) (Mitraka *et al.*, submitted), (ii) and update of the malaria ontology (IDOMAL - Infectious Disease Ontology MALaria) (19,20) and (iii) an ontology describing the domain of microRNAs (MiRNAO – MicroRNA Ontology). All VectorBase ontologies follow the rules established by the OBO (Open Biomedical Ontologies) Foundry (21), and can be browsed either at VectorBase (https://www.vectorbase.org/ontology-browser) or the NCBO (National Center for Biomedical Ontology) Bioportal (http://bioportal.bioontology.org).

### Transcriptomes (RNA-seq) and gene expression data

Advances in the rapid generation of transcriptome data using inexpensive NGS sequencing approaches (RNA-seq) have led to a proliferation of data available for invertebrate vectors. VectorBase currently presents summaries that are annotated using relevant ontologies for developmental stages, anatomy and other aspects of the experimental design (https://www.vectorbase.org/rna-seq-data-sets). We also align relevant data extracted from the Sequence Read Archive using standard alignment workflows to present coverage plots (bigWig format) via the genome browser. Customization of these displays allows for aggregation of related data into logical groups in the browser, i.e. time courses or body atlas-style studies.

More recently, we have started to quantify transcript expression values (FPKM) from these RNA-seq data based on the canonical gene set as reference. The association of mapped reads to transcripts is dynamic because of potential gene structure updates. To update and maintain mappings across assembly or gene set updates, Galaxy workflows are used.

Previously, the Expression Browser housed only data from microarray experiments. We have since upgraded the Expression Browser to facilitate the analysis of both microarray and RNA-seq experiments. These expression data continue to be managed through a third-party open source data management system called BASE (22), with the upgrade to BASE version 3.3 adding the capability to handle RNA-seq data. This necessitated upgrading our Java API (GESOL) layer that operates on top of the BASE API and simplified its data model. Our webcode has also been updated to reflect the new data classes (e.g. referring to ‘probes’ for microarray data and ‘reference transcripts’ for RNA-seq studies). As of the August 2014 release, users have access to 65 microarray experiments for *A. gambiae, A. aegypti, C. quinquefasciatus* and *Anopheles funestus*, and one RNA-seq experiment for *A. aegypti* (23) within the Expression Browser. As before, there is a requirement for data to come from replicated experiments in order to be compatible with our statistical treatment. The mix of microarray and RNA-seq data has been carried downstream into the Expression Maps, BioMart, data file downloads and site search.

### Pathways

**Figure 2.**
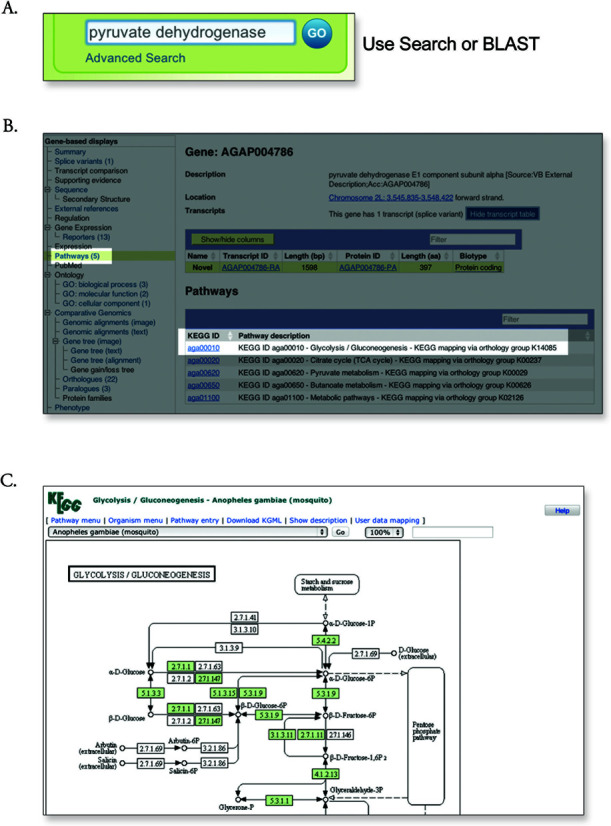
A search workflow to find genes involved in metabolic pathways. (**A**) To find a gene of interest, either search or BLAST can be used. With search, the keywords can be the gene ID (e.g. AGAP004786), the gene name (e.g. pyruvate dehydrogenase) or the gene symbol (not available for this example). If using BLAST, click on the best transcript or protein hit and follow the ‘Browse Genome’ icon to the genome browser. (**B**) In the genome browser gene tab, there will be a link called ‘Pathways’. Click on this link to load the pathways table. Follow the link in the top hit, ‘aga00010’. (**C**) This is the glycolysis/gluconeogenesis cycle in the KEGG pathways. The notation and color coding of the pathways are described in the ‘Help’ link located in the top right-hand side.

To aid in downstream analysis, pathway information from the Kyoto Encyclopedia of Genes and Genomes (KEGG) database was added for mosquitoes (*A. gambiae, A. aegypti and C. quinquefasciatus*), tick (*I. scapularis*) and lice (*P. humanus*). As shown in Figure 2, these new data are accessible from the VectorBase genome browser.

## OUTREACH

VectorBase also assists the community with a helpdesk system at info@vectorbase.org or via our ‘Contact Us’ link (https://www.vectorbase.org/contact). Helpdesk inquiries can be of any type, and we supplement our availability with online documentation such as FAQs, a Glossary of relevant terms and data policies. Constantly updated VectorBase tutorials provide training material for both novice and advanced users, and include practice exercises and sample files. Tutorials are updated several times per year. Additional outreach activities are posted on the main BRC page, and we hold *ad hoc* virtual workshops on request.

## FUTURE DEVELOPMENTS

In this update we described improvements to the existing features and integration of new data sets in terms of data integration, through search and new tools such as Web Apollo and Galaxy, along with multiple enhancements to previously reported VectorBase features. The most significant goals for the next five years are to continue to integrate reference genetic data with variation and insecticide-resistance phenotypes provided by our vector community. By becoming the NIAID-supported insecticide-resistance database, we expect to start helping our users connect genotypes to phenotypes with these and other surveillance data. Using Galaxy as a resource facilitates common and transparent analysis of these data, and VectorBase is currently developing RNA-seq and variant discovery pipelines for our users. We anticipate a mature cross-BRC workspace environment will be also developed, allowing more direct connections between the vector host and the pathogens they transmit.
